# Gastric volvulus following Nissen fundoplication: a case report and review of reported cases, risk factors, and management strategies

**DOI:** 10.1093/jscr/rjag902

**Published:** 2026-10-07

**Authors:** Nishchal Bogati, David Buchin, Andrew Johnson Godwin, Kaleab Ashebir Debebe, Minjae Kim, Rollin William Johnson

**Affiliations:** Department of Surgery, Wyckoff Heights Medical Center, 374 Stockholm St, Brooklyn, NY 11237, United States; Department of Surgery, Huntington Hospital, 270 Park Ave, Huntington, NY 11743, United States; Department of Surgery, Huntington Hospital, 270 Park Ave, Huntington, NY 11743, United States; Department of Surgery, Wyckoff Heights Medical Center, 374 Stockholm St, Brooklyn, NY 11237, United States; Department of Surgery, Wyckoff Heights Medical Center, 374 Stockholm St, Brooklyn, NY 11237, United States; Department of Surgery, Wyckoff Heights Medical Center, 374 Stockholm St, Brooklyn, NY 11237, United States

**Keywords:** gastric volvulus, organoaxial gastric volvulus, Nissen fundoplication, gastropexy

## Abstract

Gastric volvulus is a rare but potentially life-threatening complication following Nissen fundoplication, with delayed diagnosis increasing the risk of gastric ischemia, necrosis, and perforation. We report the case of a 72-year-old man who presented on postoperative Day 10 with progressive dysphagia, severe epigastric pain, and intolerance to oral intake following robot-assisted laparoscopic Nissen fundoplication. Computed tomography and upper gastrointestinal contrast study demonstrated marked gastric distension with obstruction at the gastroesophageal junction. Urgent diagnostic laparoscopy revealed a complete retroesophageal organoaxial gastric volvulus around an intact fundoplication wrap. The patient subsequently underwent robot-assisted gastric decompression, reduction of the volvulus, takedown of the fundoplication, and posterior gastropexy. Postoperative recovery was uneventful. This case highlights the importance of maintaining a high index of suspicion for gastric volvulus in patients with persistent obstructive symptoms after fundoplication and demonstrates that prompt minimally invasive surgical intervention can prevent catastrophic complications.

## Introduction

Gastric volvulus is a rare but potentially life-threatening condition characterized by abnormal rotation of the stomach along its longitudinal (organoaxial) or transverse (mesenteroaxial) axis. Acute gastric volvulus is a surgical emergency; delayed diagnosis may result in ischemia, necrosis, perforation, and hemodynamic instability [1–4]. Although most commonly associated with paraesophageal hernias and diaphragmatic defects, gastric volvulus is an exceptionally uncommon complication following Nissen fundoplication [1, 2, 5]. Its presentation often resembles expected postoperative symptoms, so diagnosis may be delayed, emphasizing the importance of a high index of suspicion. We report a rare case of acute retroesophageal organoaxial gastric volvulus around an intact fundoplication, managed with diagnostic laparoscopy followed by robot-assisted reduction, fundoplication takedown, and posterior gastropexy.

## Case report

A 72-year-old man with coronary artery disease status post angioplasty, arrhythmia, hypertension, hyperlipidemia, type 2 diabetes, and reflux disease with a hiatal hernia underwent robot-assisted laparoscopic Nissen fundoplication. His immediate postoperative course was unremarkable, and he was discharged on postoperative Day 1. On postoperative Day 10, he presented with progressive dysphagia, severe postprandial epigastric pain, recurrent vomiting, and inability to tolerate oral intake, describing food lodging in the distal esophagus, with pain relieved by emesis. He was hemodynamically stable apart from systolic hypertension (172 mmHg), with mild distension and epigastric tenderness.

Computed tomography (CT) of the abdomen with intravenous contrast showed expected postoperative changes, moderate distal esophageal distension, and marked gastric distension with an abrupt transition to a collapsed antrum, raising concern for volvulus (Fig. 1). An upper GI contrast study showed persistent contrast retention in the distal esophagus without passage into the stomach (Fig. 2). Esophagogastroduodenoscopy (EGD) revealed severe edema, ulceration, and tight narrowing at the gastroesophageal junction that even an ultraslim endoscope could not traverse (Fig. 3).

**Figure 1:**
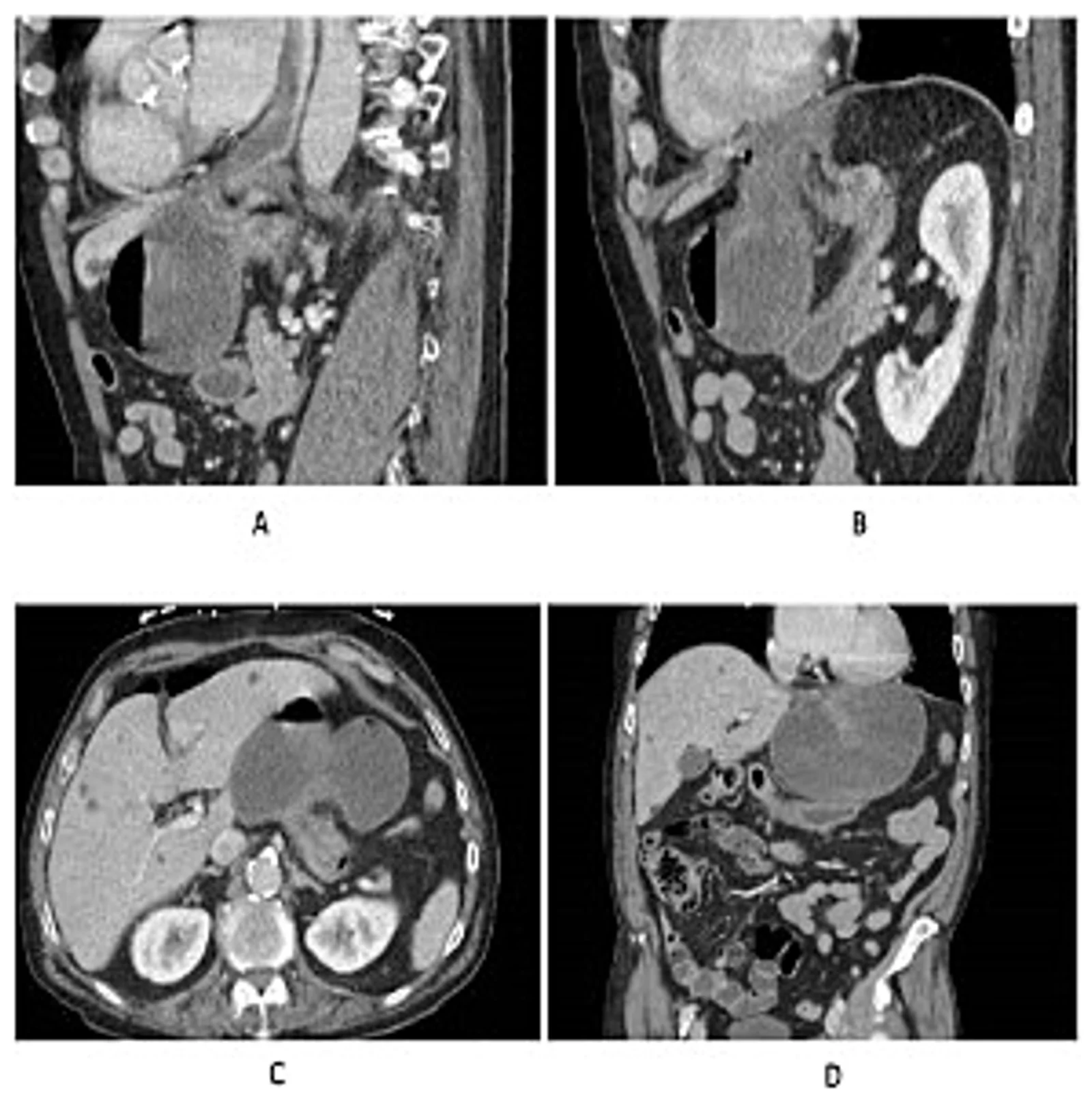
CT abdomen with intravenous contrast. (A) Dilated, fluid-filled esophagus and stomach with tapering at the hiatus. (B) Distended gastric body transitioning to a collapsed antrum. (C) Marked gastric body distension with abrupt antral collapse. (D) Antrum extending to the normally positioned pylorus and duodenal bulb.

**Figure 2:**
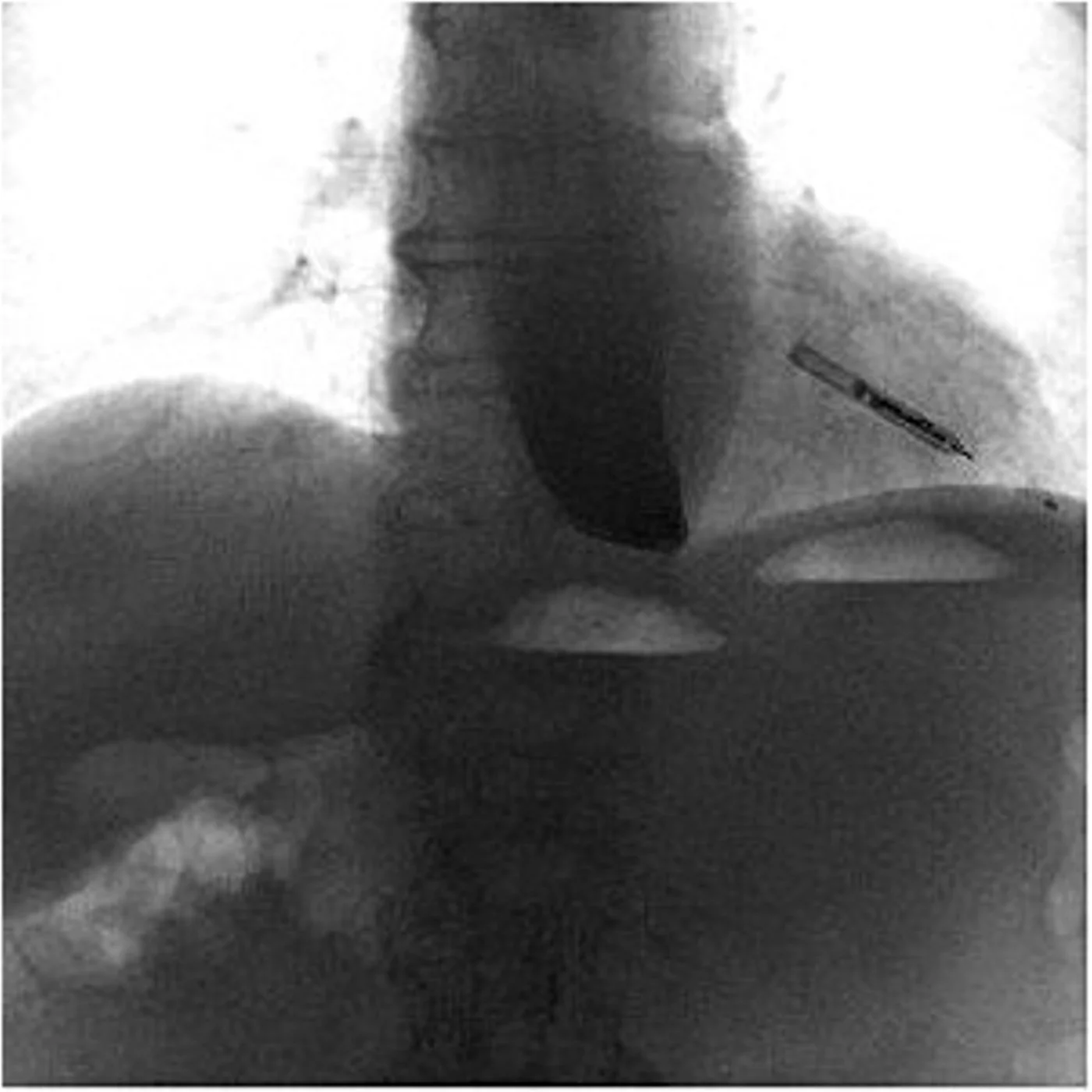
Upper gastrointestinal contrast study demonstrating retained contrast above the gastroesophageal junction without passage into the stomach**.**

**Figure 3:**
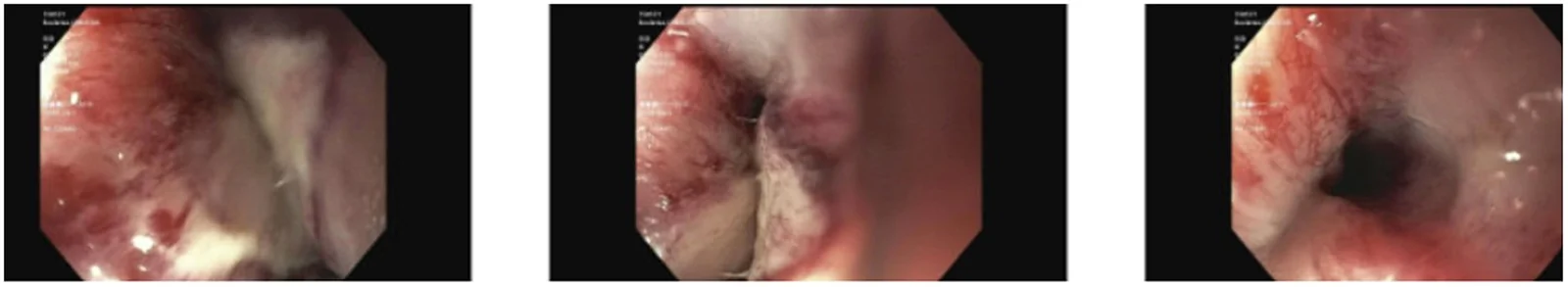
EGD demonstrating severe gastroesophageal junction erythema, stricture, and ulceration.

The patient underwent urgent diagnostic laparoscopy with robot-assisted exploration. The stomach was markedly distended, obscuring the gastroesophageal junction and fundoplication, while the distal stomach, pylorus, and duodenum remained normally positioned. A small gastrostomy was created for decompression; the gastric body was then found herniating posteriorly through the retroesophageal window, consistent with complete organoaxial volvulus around an intact fundoplication (Fig. 4).

**Figure 4:**
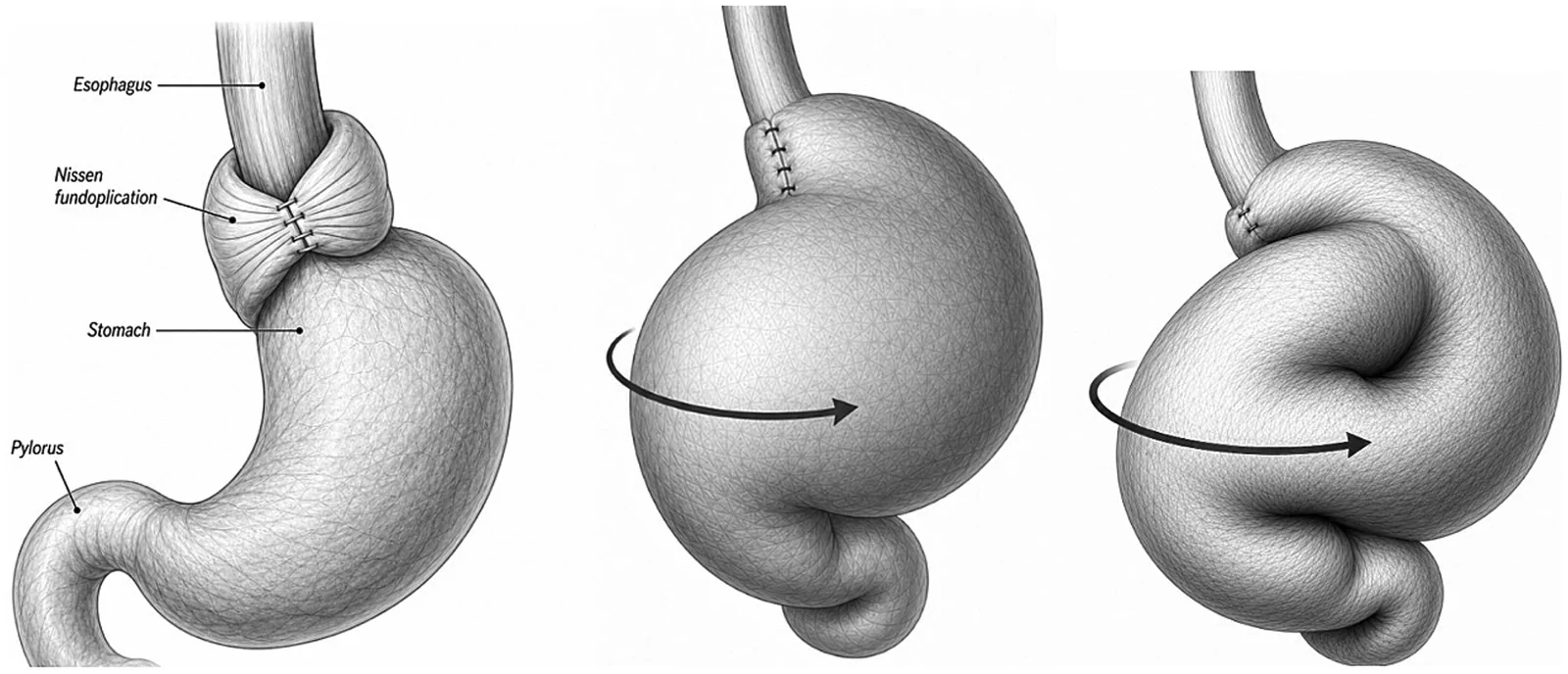
Schematic illustration depicting the intraoperative findings. (A) Intact Nissen fundoplication with a normally positioned pylorus. (B) Progressive retroesophageal rotation of the gastric body around the intact fundoplication. (C) Complete organo-axial gastric volvulus with marked gastric distension obscuring the intact fundoplication.

The stomach was reduced through the retroesophageal window, restoring normal orientation; the fundoplication was taken down to assess viability. Intraoperative endoscopy identified a few superficial gastric ulcers distal to the angle of His without transmural necrosis. Indocyanine green fluorescence imaging revealed reduced perfusion in a few focal areas of the gastric body and near the angle of His; these were reinforced with interrupted imbricating sutures. Given the acute volvulus and recurrence risk, the fundoplication was not recreated; a posterior gastropexy was performed instead. Postoperative contrast study showed no leak or obstruction; recovery was uneventful, with diet advancement before discharge.

## Discussion

Gastric volvulus is a rare but potentially life-threatening complication following Nissen fundoplication [1, 2]. Although fundoplication is well established for reflux disease and hiatal hernia, postoperative alterations in gastric anatomy and fixation may rarely predispose to abnormal rotation [1, 3]. The literature consists almost exclusively of isolated case reports [2–4]. Presentations range from intermittent obstructive symptoms to acute outlet obstruction, ischemia, necrosis, and perforation, underscoring the importance of early recognition and prompt intervention [3, 5, 6].

Our review of previously reported cases (Table 1) identified gastric volvulus after various anti-reflux and hiatal hernia repairs, with onset ranging from 2 months to 5 years postoperatively. Despite heterogeneous presentations, most patients required surgical intervention, with favorable outcomes when diagnosis was prompt and treatment preceded irreversible ischemia.

**Table 1:** Review of reported cases of gastric volvulus following fundoplication.

**Study**	**Age/Sex**	**Index surgery**	**Presentation**	**Diagnosis**	**Interval**	**Operative findings**	**Predisposing factor**	**Management**
Golash 2005 [1]	23/M	Lap Nissen	Severe upper abdominal & chest pain, retching, distension	CT: 2 air–fluid levels; gastroscopy	1 yr	Intrathoracic volvulus, adhesions, left crus rupture, caudate lobe herniation	Crus rupture, adhesions	Lap reduction, adhesiolysis, sac excision, redo Nissen + mesh, anterior gastropexy
Baty *et al*. 2002 [2]	38/M	Lap Nissen	Acute abdominal pain, vomiting	EGD: early gastric ischemia	8 mo	Volvulus from thick adhesion between xiphoid cannula site & hilus hepatis	Adhesions	Failed endoscopic detorsion → laparotomy & adhesiolysis
Vasconcelos *et al*. 2025 [3]	53/F	Lap HH repair + Nissen	Pain, nausea, vomiting	CT: mesenteroaxial volvulus; EGD torsion	1 yr	Near-complete mobilization of greater curvature; adhesion between wrap & right liver	Adhesions	Diagnostic lap → open reduction, adhesiolysis, anterior gastropexy
Albandar *et al*. 2025 [4]	36/M	Lap HH repair + Nissen	Epigastric/LUQ pain, vomiting, obstipation	CT: intrathoracic stomach; organoaxial volvulus, pleural effusion	3 yrs	Entire stomach migrated into chest; organoaxial volvulus; ischemic stomach	Diaphragmatic defect	Open gastrectomy + RYGB, defect repair, chest tube
Namata *et al*. 2024 [5]	28/M	Lap Nissen	Severe epigastric pain, distension, inability to vomit	CT: pneumoperitoneum, dilated stomach	10 mo	Organoaxial volvulus (180°), necrosis along greater curvature, perforation	Etibhond sutures as foreign body	Ex-lap, sleeve gastrectomy, anterior gastropexy
Gill *et al*. 2024 [6]	74/F	HH repair + Nissen	Abdominal pain, nausea, vomiting	CT: volvulus; UGI: 90° rotation	5 yrs	Herniation of gastric body under antrum/pylorus; mesenteroaxial volvulus under absent gastrohepatic ligament	Absent gastrohepatic ligament; over-mobilized greater curvature	Lap reduction, G-tube gastropexy, pyloric Botox injection
Savolainen *et al*. 2015 [7]	60/F	HH repair	Vomiting, severe epigastric pain, nausea	CT: massive stomach, suspected volvulus	4 mo	Volvulus without adhesions	Ligamentous laxity	Lap reduction → open hiatus repair & peritoneal fixation
Rajkumar *et al*. 2017 [8]	43/M	Lap Nissen	Acute abdominal pain, retching	CT: organoaxial volvulus	14 mo	Hiatal repair disruption; anterior adherent under gastrohepatic ligament gap	Adhesions	Lap hiatus repair, closure of pars flaccida gap, sham GJ
Chang *et al*. 2024 [9]	75/F	RA Nissen	Acute abdominal pain, PO intolerance	CT: subdiaphragmatic volvulus; EGD ischemic foci	6 mo	Torsed stomach; adhesions at fundoplication stitch–crus junction	Adhesions	Lap adhesiolysis, takedown, reduction, gastropexy
Reyes-Zamorano 2014 (Case 1) [10]	29/F	Lap Nissen	Severe epigastric hematemesis, PO intolerance	EGD bleeding; UGI organoaxial volvulus	4 mo	Organoaxial volvulus; fibrotic band from gastrocolic ligament to wrap; wrap fixed to right crus	Adhesions	Lap reduction, gastrorrhaphy, redo 270° wrap, gastropexy
Reyes-Zamorano 2014 (Case 2) [10]	40/F	Lap Nissen	Severe epigastric distension, vomiting	UGI: mesenteroaxial volvulus	6 mo	Mesenteric torsion; pylorus/antrum/omentum passed through wrap → trans-fundoplication hernia	Trans-fundoplication internal hernia	Lap detorsion, redo Nissen, closure of defect
Suwal *et al*. 2020 [11]	57/F	Lap diaphragm repair + Nissen	Acute dysphagia, epigastric pain	CT: intrathoracic wrap migration; EGD tight wrap, mucosal ischemia	7 mo	Incarcerated intrathoracic wrap with necrotic fundus	Intrathoracic wrap migration	Lap subtotal gastrectomy, mediastinal drainage, defect closure
Koh *et al*. 2011 [12]	81/F	Lap fundoplication + mesh	Acute abdominal pain	CT: perforated mesenteroaxial volvulus	2 mo	Perforated mesenteroaxial volvulus	Not specified	Laparotomy, gastrectomy
Chattopadhyay *et al*. 2011 [13]	38/F	Lap Nissen	Upper abdominal persistent vomiting	EGD: paraesophageal hernia; CT: distended esophagus, displaced fundus	3 mo	Cephalad internal herniation of anterior gastric body under attenuated wrap	Trans-fundoplication internal hernia	Lap reduction, Toupet fundoplication

### Mechanism of postoperative gastric volvulus

Pathogenesis is likely multifactorial, including postoperative adhesions, recurrent paraesophageal herniation, intrathoracic migration of the stomach or wrap, trans-fundoplication internal herniation, and laxity of gastric ligamentous attachments [1–4, 6–8]. Adhesions were the most frequently reported finding, creating fixed points around which the stomach rotated; recurrent herniation and intrathoracic migration were also common with organoaxial volvulus [2, 4, 6]. Increased gastric mobility after fundal mobilization and short gastric vessel division may diminish ligamentous restraint and, with acute distension as a precipitant, permit pathological rotation.

In the present case, complete retroesophageal organoaxial volvulus developed around an intact fundoplication without recurrent hernia, wrap disruption, or intrathoracic migration. We propose the intact wrap acted as a fixed fulcrum while increased gastric mobility and acute distension triggered volvulus.

### Diagnosis

Diagnosis remains challenging because symptoms—nausea, vomiting, dysphagia, epigastric pain, distension, and oral intolerance—overlap with expected postoperative complaints, delaying recognition [2–4, 7]. Delay increases risk of ischemia, necrosis, perforation, and need for resection [5–7]. CT is the preferred initial modality, rapidly demonstrating abnormal gastric orientation, outlet obstruction, recurrent herniation, and features of ischemia [3, 4, 6]. Upper GI contrast studies complement CT by confirming obstruction or volvulus [3, 4, 6]. EGD adds value by excluding mechanical obstruction and assessing mucosal viability, but should not delay definitive imaging and surgery [9–11, 13].

### Operative management

Treatment depends on acuity, anatomic abnormality, and gastric viability, generally involving reduction of the volvulus, correction of the defect, repair of recurrent hernia when present, and restoration of fixation via gastropexy, with or without wrap revision [1, 3, 4]. Delayed diagnosis or ischemia may necessitate gastrectomy [5–7]. In our case, the stomach remained viable, permitting successful laparoscopic reduction. The fundoplication was taken down to assess viability; since the wrap was intact with no recurrent hernia, redo fundoplication was deferred given concern for recurrent herniation. Posterior gastropexy was performed instead.

### Gastropexy and clinical implications

Routine gastropexy is not recommended after Nissen fundoplication for uncomplicated reflux disease, since the wrap provides fixation and postoperative volvulus is exceedingly rare [2]. It is reserved for high-risk scenarios—large paraesophageal hernia repair, recurrent or concurrent volvulus, and unfavorable anatomy such as >30% gastric herniation or a crural defect >4 cm [14]. A recent review similarly concluded gastropexy should be used selectively, not routinely, in most paraesophageal hernia repairs [15].

## Conclusion

Gastric volvulus following Nissen fundoplication is a rare but potentially life-threatening complication requiring a high index of suspicion, as its presentation often mimics expected postoperative symptoms. Prompt diagnosis with CT and timely surgical intervention are essential to prevent ischemia, necrosis, and other complications. This case highlights a unique mechanism of retroesophageal organoaxial volvulus around an intact fundoplication, demonstrating that early recognition and appropriate management yield excellent outcomes.
